# Adequacy of Critical Nutrients Affecting the Quality of the Spanish Diet in the ANIBES Study

**DOI:** 10.3390/nu11102328

**Published:** 2019-10-01

**Authors:** Josune Olza, Emilio Martínez de Victoria, Javier Aranceta-Bartrina, Marcela González-Gross, Rosa M. Ortega, Lluis Serra-Majem, Gregorio Varela-Moreiras, Ángel Gil

**Affiliations:** 1 Department of Biochemistry and Molecular Biology II, Institute of Nutrition and Food Sciences, University of Granada, Campus de la Salud, Avda. del Conocimiento, Armilla, 18100 Granada, Spain; jolza@ugr.es; 2 Instituto de Investigación Biosanitaria ibs.Granada, 18100 Granada, Spain; 3 CIBEROBN, Biomedical Research Networking Center for Physiopathology of Obesity and Nutrition, Carlos III Health Institute, 28029 Madrid, Spain; javieraranceta@hotmail.com (J.A.-B.); marcela.gonzalez.gross@upm.es (M.G.-G.); lluis.serra@ulpgc.es (L.S.-M.); 4 Department of Physiology, School of Pharmacy Institute of Nutrition and Food Sciences, University of Granada, Campus de la Salud, Avda. del Conocimiento, Armilla, 18100 Granada, Spain; emiliom@ugr.es; 5 Department of Preventive Medicine and Public Health, University of Navarra, c/Irunlarrea 1, 31008 Pamplona, Spain; 6 ImFINE Research Group, Department of Health and Human Performance, Universidad Politécnica de Madrid, c/Martín Fierro 7, 28040 Madrid, Spain; 7 Department of Nutrition and Food Science, Faculty of Pharmacy, Madrid Complutense University, Plaza Ramón y Cajal s/n, 28040 Madrid, Spain; rortega@ucm.es; 8 Research Institute of Biomedical and Health Sciences, University of Las Palmas de Gran Canaria (IUBS), and Service of Preventive Medicine, Complejo Hospitalario Universitario Insular Materno Infantil (CHUIMI), Canary Health Service, 35016 Las Palmas de Gran Canaria, Spain; 9 Spanish Nutrition Foundation (FEN), 28010 Madrid, Spain; gvarela@ceu.es; 10 Department of Pharmaceutical and Health Sciences, Faculty of Pharmacy, CEU San Pablo University, Urb. Montepríncipe, Crta. Boadilla Km 53, Boadilla del Monte, 28668 Madrid, Spain

**Keywords:** adequacy of intake, diet quality, diet quality indexes, dietary recommended intakes, nutrients

## Abstract

Diet is one of the key modifiable behaviors that can help to control and prevent non-communicable chronic diseases. Therefore, it is important to evaluate the overall diet composition of the population through non-invasive and independent indexes or scores as diet quality indexes (DQIs). The primary aim of the present work was to estimate the adequacy of the intake of critical nutrients in the Spanish “Anthropometry, Intake, and Energy Balance Study” (ANIBES) (*n* = 2285; 9–75 years), considering, as a reference, the European Food Scientific Authority (EFSA) values for nutrients for the European Union. We also assessed the quality of the diet for adults and older adults using four internationally accepted DQIs, namely the Healthy Diet Indicator (HDI), the Mediterranean Diet Score (MDS), the Mediterranean Diet Score-modified (MDS-mod), and the Mediterranean-Diet Quality Index (MED-DQI), as well as the ANIBES-DQI, stratified by education and income. The ANIBES-DQI was based on compliance with EFSA and Food and Agriculture Organization recommendations for a selected group of nutrients (i.e., total fat, saturated fatty acids (SFAs), simple sugars, fiber, calcium, vitamin C, and vitamin A), with a total range of 0–7. Misreporting was assessed according to the EFSA protocol, which allowed us to assess the DQIs for both the general population and plausible reporters. The majority of the Spanish population had high intakes of SFAs and sugars and low intakes of fiber, folate, and vitamins A and C. In addition, about half of the population had low DQI scores and exhibited low adherence to the Mediterranean diet pattern. Overall, older adults (>65–75 years) showed better DQIs than adults (18–64 years), without major differences between men and women. Moreover, primary education and low income were associated with low MDS and ANIBES-DQI scores. For the ANIBES-DQI, the percentage of the population with low scores was higher in the whole population (69.5%) compared with the plausible energy reporters (49.0%), whereas for medium and high scores the percentages were higher in plausible reporters (41.2% vs. 26.2% and 9.8% vs. 4.3%, respectively). In conclusion, the present study adds support to marked changes in the Mediterranean pattern in Spain, and low education and income levels seem to be associated with a low-quality diet. Additionally, the misreported evaluation in the ANIBES population suggests that this analysis should be routinely included in nutrition surveys to give more precise and accurate data related to nutrient intake and diet quality.

## 1. Introduction

Non-communicable diseases (NCDs), such as cardiovascular diseases, type 2 diabetes, cancer, respiratory diseases, and other inflammatory pathologies, represent approximately 70% of deaths worldwide [1]. This group of diseases shares common risk factors, namely unhealthy diets, sedentariness and low physical activity, excess consumption of alcohol, and the use of tobacco products, all of which are risk factors for obesity, hypertension, and lipid and glucose metabolism impairment that lead to the establishment of chronic diseases [2].

As diet is one of the critical modifiable behaviors that can help control cardiometabolic risks and prevent chronic diseases, it is essential to evaluate the overall diet composition of the population [3,4]. Diet quality indexes (DQIs) are tools that aim to evaluate the quality of diets and categorize individuals according to the extent to which their eating behavior is “healthy” [5]. 

Currently, there is a large number of DQIs, most of which are designed or adapted to reflect the nutritional needs of different population groups and adherence to specific dietary patterns [5,6]. DQIs are comprised of various foods and/or nutrients to assess overall diet quality [7]. There are three major categories of DQIs: (a) Nutrient-based indicators, (b) food/food group-based indicators, and (c) combination indexes. Thus, each DQI has different foods and nutrients components, cut-offs, and scoring approaches, which limits comparability. Moreover, most DQIs have been developed for specific populations. Indeed, even though DQIs can be used in similar groups of people, in most cases, their use is limited to populations different from those for which they were specifically designed. Therefore, it is challenging to compare the results among studies that use different DQIs. 

The Healthy Diet Indicator (HDI) and the Mediterranean Diet Score (MDS) are within the four “original” diet quality scores that have been referred to and validated most extensively [5,6,7,8]. Additionally, several DQIs have been adapted and modified from those originals. In particular, many variations on the MDS have been proposed, and the so-called “MDS modified” (MDS-mod) and the Mediterranean-Diet Quality Index (MED-DQI) have been widely used among Mediterranean populations. The latter has been reported to be one of the most adequate scores to evaluate the Mediterranean diet in adults [5]. However, these scores do not necessarily capture what is needed for European Union (EU) populations, as in 2017, the European Food Safety Authority (EFSA) published the dietary reference values for nutrients for the EU [9]. Thus, a new DQI considering the EFSA nutrient recommendations would be useful in EU nutrition surveys.

DQIs have been reported to be influenced by socioeconomic determinants, mainly education and income. [10]. However, the majority of studies do not control for these covariates, which limits comparability. Additionally, most epidemiologic data are collected through surveys, so self-report biases and misreporting are possible [11]. For this reason, the EFSA has published a protocol that has a harmonized approach to identify misreporting [12]. Following this protocol, we previously reported the intake of energy, some minerals, and vitamins for both the Spanish “Anthropometry, Intake, and Energy Balance Study” (ANIBES) in the general population and plausible reporters [13].

Considering the above, the primary aim of the present study was to evaluate the adequacy of critical nutrients that directly affect the quality of the diet of the Spanish ANIBES population, stratified by sex and different age groups, using the EFSA values for nutrients for the EU as a reference. Second, we assessed the quality of the diet for adults and older adults using HDI, MDS, MDS-mod, and MED-DQI, as well as a new score, ANIBES-DQI, stratified by education and income. The ANIBES-DQI is based on compliance with EFSA recommendations for a selected group of nutrients (i.e., total fat, saturated fatty acids (SFAs), simple sugars, fiber, calcium, vitamin C, and vitamin A (total range 0–7)). One additional aim was to assess the above mentioned DQIs for both the general ANIBES population and plausible reporters.

## 2. Materials and Methods

### 2.1. Study Design and Participants 

The ANIBES study design, protocol, and methodology have been described in detail elsewhere [14]. Briefly, it is a cross-sectional study conducted using multistage stratified sampling. The fieldwork was performed at 128 sampling points across Spain. The sample consisted of 2285 participants aged 9–75 years (1160 men and 1125 women), of which 2009 were randomly selected (1013 men, 996 women), and 176 participants came from a “booster sample” to provide at least 200 individuals for the youngest (9–12, 13–17, and 18–24 years) and oldest (65–75 years) age groups. The sample quotas were distributed by age, sex, geographical distribution, and locality size [14,15]. In addition, other data related to factors such as the unemployment rate, the percentage of non-Spanish subjects, physical activity level, and education and income levels were collected.

### 2.2. Dietary Data Collection

The availability of detailed, harmonized, and high-quality food consumption data for use in dietary exposure assessments is a long-term objective of EFSA. The 2014 updated guidance document covers the EU’s Menu methodology and therefore facilitates the collection of more harmonized food consumption data from all EU Member States [16]. Briefly, for the 24 h dietary recall (24HR), a computer-assisted personal or telephone interview (CAPI/CATI) method should be used, and the reported foods should be described in accordance with the EFSA FoodEx2 food classification system. Indeed, study participants were provided with a tablet device (Samsung Galaxy Tab 2 7.0, Samsung Electronics, Suwon, Korea) to register their food consumption over the course of three days [14]. Participants that were unable to use the tablet device were offered other options, such as using a digital camera, a paper record, or telephone interviews. Reported intakes for food, beverages, and energy and nutrients were calculated from food consumption records using the VD-FEN 2.1 software, a Dietary Evaluation Program from the Spanish Nutrition Foundation (FEN). The program was newly developed for the ANIBES study by the FEN and is based mainly on Spanish food composition tables [17]. Data obtained from food manufacturers and nutritional information provided on food labels were also included. A photographic food atlas was used to assist in assigning gram weights to portion sizes [15]. 

Physical activity was also assessed during interviews with the international physical activity questionnaire (IPAQ). For a 10% subsample, physical activity was also determined using accelerometers. The details are published elsewhere [18].

### 2.3. Adequacy of Reported Intake 

The average requirements (ARs) published by the EFSA, according to age and sex cut-points, were applied to estimate the prevalence of adequate protein, calcium, iron, zinc, folate, vitamin C, and vitamin A intake [9]. The AR is the level of nutrient intake that is adequate for half of the people in a population group, given a normal distribution of requirements [9]. When there is no population reference intake (PRI) to establish it, adequate intake (AI) is used instead [9]. AI is the observed daily average intake by a population group (or groups) of apparently healthy people that is assumed to be adequate. In the case of fiber, we compared the reported intake with the EFSA’s AI. For lipids and sugars, we followed the Food and Agriculture Organization (FAO) Fats and Fatty Acids in Human Nutrition Report recommendations [19] and the World Health Organization (WHO)’s guidelines [20], respectively. 

### 2.4. Diet Quality Scores

The four previously developed DQIs calculated were HDI [21], MDS [22], MDS-mod [23], and the MED-DQI [24]. Children and adolescents were excluded from this analysis, as the DQI scores used in the present work were defined for adult populations. Table 1 summarizes the characteristics of each score.

#### 2.4.1. HDI

The HDI was selected because it has been used in many studies worldwide to evaluate dietary patterns using the WHO guidelines for the prevention of chronic diseases. A dichotomous variable was generated for each food group or nutrient that was included in these guidelines. If a person’s intake was within the recommended range, this variable was coded as 1. Otherwise, it was coded as 0. The HDI was the sum of all these dichotomous variables, including SFAs, polyunsaturated fatty acids (PUFAs), cholesterol, protein, complex carbohydrates, monosaccharides and disaccharides, dietary fiber, fruits and vegetables, pulses, nuts, and seeds [21]. For the HDI, the cut-off values were <3, 3–4, and >4, categorized as low, medium, and high, respectively.

#### 2.4.2. MDS

The traditional Mediterranean diet has been defined and reasonably scored in terms of eight component characteristics: A high monounsaturated to saturated fat ratio, moderate ethanol consumption, high consumption of legumes, high consumption of cereals (including bread and potatoes), high consumption of fruits, high consumption of vegetables, low consumption of meat and meat products, and low consumption of milk and dairy products [22]. A value of 0 or 1 was assigned to each of eight components with the use of the sex-specific median as the cut-off, total range 0–8. The MDS cut-off was 4. Scores <4 were classified as low and those ≥4 as high.

#### 2.4.3. MDS-Mod

This score is a revised scale of the MDS, including fish intake as part of the degree of adherence to the traditional Mediterranean diet [23]. A value of 0 or 1 was assigned to each of nine components with the use of the sex-specific median as the cut-off, total range 0–9. The MDS-mod cut-off values were <4, 4–5, and >6, corresponding to low, medium, and high diet quality, respectively.

#### 2.4.4. MED-DQI

This index includes the intake of SFAs and cholesterol and the consumption of meats, olive oil, fish, cereals, and vegetables plus fruits [24]. Each nutrient or food group was assigned three scores (0, 1, and 2) on the basis of the recommended guidelines, where such guidelines exist (cholesterol, SFA), or by dividing the population’s consumption into tertiles, where there was no specific recommendation for the food [5,24]. The MED-DQI scores were totaled as follows: Good, 1–4; medium–good, 5–7; medium–poor, 8–10; poor, 11–14. 

### 2.5. ANIBES-DQI

A group of critical nutrients related to diet, namely proteins, total fat, SFAs, PUFAs, simple sugars (including sugars naturally found in foods and added sugars), fiber, calcium, iron, zinc, folate, vitamin C, and vitamin A, were initially considered to evaluate whether their intakes were adequate on a population level. Later, a selected group of those nutrients (i.e., total fat, SFAs, simple sugars, fiber, calcium, vitamin C, and vitamin A) were grouped to create the “ANIBES-DQI”, potentially applicable to any EU population. Iron was not included, as the majority of the ANIBES population had an adequate intake of this micronutrient. Folate was not included, as its intake exhibited a high co-linearity with both vitamin C and fiber. Finally, zinc was not included, as the database food composition used in the present study lacks some data for this micronutrient in various foods usually consumed by adults. 

Each of the criteria was scored as either 0 (not compliant) or 1 (compliant) based on the EFSA dietary reference for nutrients [9], the FAO Fats and Fatty Acids in Human Nutrition Report recommendations for lipids [19], and the WHO guidelines for sugars [20]. The ANIBES-DQI ranged from 0 to 7. Cut-off values were <3, ≥3 to <5, and >5: low, moderate, and good, respectively. The criteria to score either 0 or 1 for each component are shown in Table 2. 

### 2.6. Covariates

Participants were categorized according to primary education, secondary education, and tertiary education based on the International Standard Classification of Education (ISCED) scale [25]. The income of participants was grouped in three categories: <1000, 1000–2000, and >2000 Euros/month, corresponding to low medium, and high income, respectively.

### 2.7. Misreporting

The methodology used in the evaluation of misreporting for the ANIBES population has been reported previously [13]. Briefly, the EFSA protocol to calculate misreporting was followed [12]. This protocol is mainly based on the studies of Goldberg [26] and Black [27,28]. This method compares the reported energy intake (EIrep) with the presumed energy expenditure. EIrep is expressed as a multiple of the estimated mean basal metabolic rate (BMRest). Then, the ratio EIrep:BMRest is referred to as the physical activity level (PAL) [12]. The PAL is established for youth (≤17 years) and adults (18-65 years) in three levels: Low (1.6 and 1.4), moderate (1.8 and 1.6), and vigorous (2.0 and 1.8). Additionally, the protocol indicates that analyses should be done at two levels: Group and individual. The group level determines the overall bias to the reported EI, and the individual level shows the rate of under and over reporters. We calculated the BMRest using the Schöfield equations [29]. Misreporting cut-offs at the group and individual levels for the ANIBES study have been previously reported [13]. Briefly, the percentages of plausible reporters by age groups were adults (26%) and older adults (22%). 

As mentioned earlier in Section 2.4, in the present study, we excluded children and adolescents not because the ratio EIrep:BMRest referred to as the PAL cannot be calculated for those groups but because the DQI scores were defined for adult populations.

### 2.8. Statistical Analysis 

To evaluate the adequacy of the reported nutrient intake, we compared the so called “usual intake” with the AR or AI, published by EFSA according to age and sex. Since we average the intake of a small number of days of 24HR (observed intakes; i.e., 3 non-consecutive days in the ANIBES study do not adequately reflect the usual intake), it is necessary to statistically model the dietary data to eliminate day-to-day variation in food consumption. Thus, the usual intake distribution was estimated from the observed daily intake, applying a statistical age-dependent correction for the intra-individual (day-to-day) variation [30] using SPADE (Statistical Program to Assess Dietary Exposure, RIVM, Amsterdam, Nederlands) [31]. Briefly, this program estimates the percentiles of habitual nutrient intake distributions and the proportions above or below the defined AR or AI cut-off values. The interview day (day 1, day 2, or day 3), the day of the week, the season, and the sample weighting factor are considered in the adjustment of dietary data, stratified by sex and age group. 

The chi-squared test was used to estimate differences in the percentages of subjects classified into categories according to the cut-off values for each DQI.

Kendall’s Tau rank correlation coefficient was used to evaluate the potential association of each DQI score to education and income levels and their interactions. Given that DQI scores differed by the number of point-scale (i.e., for HDI, low, medium, and high; for MDS, low and high; for MDS-mod, low, medium, and high; for MED-DQI, poor, medium-poor, medium-good, and good; and for ANIBES-DQI, low, medium, and high), the model of analysis was based on a non-square contingency table. Indeed, for the analysis of data, we used Tau-c rank correlation coefficient statistics. 

To estimate the specific effects of the covariates “education” and “income level” (ordinal independent variables) on the degree of diet quality (i.e., categories for each DQI (ordinal dependent variables)) we used an ordinal regression Generalized Linear Model (GLM) with “logit” as the link function (PLUM procedure of Statistical Package for Social Sciences (SPSS), version 25.0). The estimated parameters are the exponential values of the regression coefficient (*x*), which give the probability for the association of specific DQI categories with a particular education or income level. That is to say, the exponential e^−*x*^ indicates the probability of significant effects of the covariates on different categories (e.g., low, medium, and high for each of the DQIs). A *p*-value <0.05 was considered significant.

## 3. Results

### 3.1. Adequacy of Critical Nutrients in the ANIBES Population

Table 3 shows the percentage of the ANIBES population with reported inadequate intake of proteins, PUFA, SFA, simple sugars, fiber, calcium, iron, zinc, folic acid, vitamin C, and vitamin A by age and sex. About 14.8% of the older adults did not reach the AR of proteins, whereas the rest of the population mostly complied with the EFSA AR. With regards to fatty acids, intake of PUFA was within the range of FAO recommendations for about 75% of the whole population, whereas saturated fatty acid intake was over the maximum recommendation for more than 50% of the population, being the largest for children (80.6%) and adolescents (76.5%). The intake of simple sugars was higher than the recommended AR (87% for adults to about 93% for children). Likewise, a high percentage of the population had a low intake of dietary fiber (85–99% of the AI recommended by EFSA). About one half of children exhibited a low intake of calcium, reaching 75.4% in adolescents. Intermediate values were observed for adults and older adults (65–68%). However, the intake of iron was close to the AR for the majority of the population (95–98%). With respect to zinc, 31.3% of the children and 76.9% of the adolescents did not comply with the EFSA AR recommendations. Data for zinc intake in adults and older adults were not included, as the database food composition used in the present study lacked some data for this micronutrient in various foods regularly consumed by the adult population. Regarding folate intake, a high percentage of the population did not reach the AR (mainly adolescents, adults, and older adults (81.8–88.3%)). The intakes of vitamin C and vitamin A exhibited similar patterns, particularly in adolescents and adults (close to 50% AR).

### 3.2. Diet Quality Indexes

Table 4 shows the DQI scores classified according to the cut-off values for the whole ANIBES population, adults (18–64 years), and older adults (65–75 years) by sex. About 60.2% of adults and 49.0% of older adults had a low HDI. However, about 10.2% of older adults exhibited a high DQI compared with only 4.8% of adults. No major differences were found between men and women.

With regards to the Mediterranean dietary adherence evaluated by MDS, 47.4% of adults and 23.8% of older adults had a low score. A similar pattern was found when MDS-mod was used. However, the absolute values were lower compared to MDS (35.5% for adults vs. 14.6% for older adults). For both indexes, older adults had a higher score compared to adults (MDS 76.2% vs. 52.6; MDS-mod 37.4% vs. 22.8%). As per the MED-DQI index, 70.8% of older adults showed a medium–good to a good score, compared to 48.2% of adults. 

The ANIBES-DQI lead to the lowest values of diet quality for both adults and older adults (70.7% and 60.2%, respectively), compared to the other DQIs. Similar scores for high dietary quality were found for HDI and ANIBES indexes (3.9% and 7.8%, respectively).

#### 3.2.1. DQIs Stratified by Education and Income 

Table 5 summarizes the percentages of the whole population for each DQI stratified by education and income levels, as well as the associations of the degree of diet quality with these covariates. Supplementary Table S1 shows the percentages of the whole population according to DQIs, stratified by education per income, and Supplementary Table S2 summarizes the major estimated effects of education and income on the degree of diet quality for each DQI (i.e., ordinal regression coefficients (*x*), the probability of significant effects of the covariates on different categories—e^−*x*^, and significance). 

There were significant associations between education, income and its interactions (education × income) with MDS. A significant association was also found between income and the interaction of income with education for the ANIBES-DQI. However, no significant associations were observed for the other DQIs

For MDS, the ordinal regression coefficient associated with primary education (0.455) was significantly different from zero (*p* < 0.001). Therefore, the exponential value of the regression coefficient associated with the education variable was e^−0.455^ = 0.634, which indicates that the probability of having a low MDS with primary education was 63.4% higher than the probability of having a low MDS with secondary education. The coefficient associated with low income (0.359) was significantly different from zero (*p* = 0.019). Thus, the exponential value of the regression coefficient associated with the income variable was e^−0.359^ = 0.698, which indicates that the probability of having a low MDS with low income was about 70% higher than the probability of having a low MDS with high income. Applying the same procedure, as described above, the probability of having a low MDS with low income was 76.5% higher than the probability of having a low MDS with middle income, and the probability of having a low MDS with primary education and middle income was 62% higher than the probability of having a low MDS with secondary education and middle income. For MDS-mod, the probability of having a low or medium score with secondary education was about 38.5% higher than having a low and medium score with tertiary education.

For MED-DQI, the probability of having a poor index with primary education was 68.6% higher than the probability of having a poor index with secondary education. Further, the probability of having a poor index with secondary education was 31% higher than the probability of having a poor index with tertiary studies. 

For the ANIBES-DQI, the probability of having a low and medium score with low income was about 48.5% higher than having a low and medium score with high income. In addition, the probability of having a low or medium score with secondary education was around 42.5% higher than having a low or medium score with tertiary education Further, the probability of having a low or medium score with secondary education and high income was about 67.5% higher than having a low or medium score with tertiary education and high income.

#### 3.2.2. DQI According to Misreporting

Supplementary Table S3 shows the percentages of the whole population and the plausible energy reporters for each DQI, stratified by age and sex.

Overall, there was a similar pattern for the total population and plausible reporters related to DQIs, except for the ANIBES-DQI, in which the percentages of the population with low scores were higher in the whole population (69.5%) compared to the plausible energy reporters (49.0%). Additionally, for medium and high ANIBES-DQI, the percentages were higher in plausible reporters (41.2% vs. 26.2% and 9.8% vs. 4.3%, respectively).

## 4. Discussion

In the present study, we evaluated the adequacy of the intake of critical nutrients related to the quality of the diet for the ANIBES study population using the EFSA dietary reference values for nutrients [9]. In addition, we assessed the quality of the diet through internationally recognized DQIs: one general index (HDI) and three related to adherence to the Mediterranean diet (MDS, MDS-mod, and MED-DQI), as the Spanish population should generally follow a Mediterranean lifestyle pattern. Likewise, we built a novel index, the ANIBES-DQI, based on the adequacy of compliance to the current WHO/FAO guidelines for total fat and fatty acids [19], the WHO guidelines for sugar intake [20], and the EFSA recommended intakes for fiber, vitamin C, vitamin A, zinc, and calcium [9]. We assessed all DQIs for both a general Spanish population and plausible reporters.

The Mediterranean diet encompasses the traditional dietary patterns found in the olive-growing regions of the Mediterranean basin in the 1960s. It is globally recognized as a healthy dietary model and an intangible cultural heritage of humanity by the United Nations Educational, Scientific and Cultural Organization (UNESCO) [32]. Food consumption patterns in Spain and energy and nutrient intakes have changed markedly in the last forty years, differing somewhat at present from the traditional and healthy Mediterranean Diet. This change is partly due to the westernization of eating habits [33,34,35].

The detailed analysis of the ANIBES population, with regard to the recommended intakes for the nutrients included in the ANIBES-DQI, shows that, except for protein, PUFA, and iron, the Spanish population have higher intakes of SFA, simple sugars, and low intakes of fiber, calcium, zinc, folate, and vitamins A and C than recommended. Overall, older adults showed better DQIs than adults, with no major differences found between men and women, although the percentages of women with a low-quality score were slightly lower than those for men. Regarding the association of DQIs with education and income, in general, education level had a strong influence on the quality of the diet, although HDI did not discriminate between different levels of education. Globally, lower education levels were related to lower quality scores for the Mediterranean diet quality indexes, regardless of the analyzed DQI. Nonetheless, using the ANIBES score, we observed a lower score for those with low income. 

When comparing the DQIs for the general population and plausible reporters, we observed similar percentages for low, medium, and high scores, except for the ANIBES-DQI, for which the percentages of the low-quality score were higher, and those with higher quality scores were lower in plausible reporters. The differences found in the percentages of the population with low quality scores for the analyzed DQIs could be due (at least in part) to the special particularities of the Spanish ANIBES population. In fact, most of the DQIs have been designed for specific populations [5]. Indeed, the MDS, MDS-mod, and MED, which measure the adherence to the Mediterranean diet, exhibited lower percentages of low-quality scores compared to the indexes potentially applicable to general populations, such as HDI and ANIBES-DQI. Some authors have reported that certain DQIs built for specific populations lead to lower quality indexes when adapted to others with different food patterns [5]. We also observed that a higher percentage of the population (approximately 70%) had a low-quality diet when using the HDI, compared to approximately 60% for the ANIBES-DQI. This difference might be attributable to the fact that HDI is a mixed quality diet index based on the consumption of specific groups of foods and the adequacy of the intake of selected nutrients, whereas the ANIBES-DQI takes into account, exclusively, the adequacy of the intake of a number of critical nutrients compared to EU reference nutrient intakes. Therefore, it seems crucial when using DQIs to consider their specific purposes, for example, whether they are used for the evaluation of dietary intakes and food patterns, the development of dietary guidelines for specific populations, or the prevention of NCDs. In fact, we have previously pointed out that there is no standardized approach to the components of DQIs and scoring, as the latter is based on food frequency, number of portions, assigned food weights, compliance with nutrient recommendations, etc. It is difficult to compare DQI scores, which are often country-specific [5]. Nevertheless, in the present study, we tried to compare whether internationally recognized DQIs exhibited a similar behavior for the Spanish population. 

When HDI, MDS, MDS-mod, and MED were compared with the ANIBES-DQI, we observed that the latter, built on nutrients exclusively, was more strict when scoring diet quality than those based on nutrients and foods. Whatever the case, it seems that the inclusion of the consumption of foods in DQIs beyond the selected critical nutrients contributes to improving the results of the quality assessment of diet in most of populations [5]. In these cases, it is very important to select and validate an appropriate method for the evaluation of food consumption (e.g., a quantitative FFQ), as well as to use a data base of quality for the composition of foods. 

Recently, the memory-based dietary assessment methods utilized in epidemiological research related to food group consumption and major events of disease have been challenged due to the varied precision and accuracy of self-reported data [36,37,38]. Archer et al. [11,36,38] empirically refuted memory-based dietary assessment methods (M-BM), such as food records, food frequency questionnaires (FFQs), and 24HR, arguing that the errors associated with M-BM-data are unquantifiable, as they are prone to omissions, false memories, intentional misreporting (i.e., lying), and gross misestimations. In particular, FFQs are prone to measurement error [39]. However, other investigators strongly disagree with the assertions made by those authors regarding the validity and usefulness of FFQs and other M-BM in assessing diet–disease relationships in epidemiologic studies. Indeed, Martin Calvo and Martinez-Gonzalez [40] emphasized that the growing evidence regarding diet–disease relationships found in observational studies based on M-BM is sufficiently reliable to be used for public health policies. In addition, well-controlled prospective studies using objective biomarkers of intake have confirmed the results of previous studies using self-reported dietary assessment methods. Regardless of this debate, it seems that to compare diverse DQIs for different populations, the methods of dietary assessment and databases for food composition would need to be harmonized. We do not think one unique central data database should be chosen, but instead that each data base for particular countries should be harmonized and based on the same methodology to help reduce the variability. In that respect, the EFSA has given specific guidelines to facilitate the collection of food consumption data from all EU Member States [16]. 

In the ANIBES study, diet was evaluated throughout a three-day dietary record using a tablet device and digital cameras to collect the information, accompanied by a 24HR [14,15]. Moreover, a group of experts supervised the trained personnel to minimize all the possible issues that could potentially appear during the data collection process. However, we are aware that there are aspects that we cannot control, such as the accuracy of memory when recording the past eating and drinking behaviors of the volunteers, which are subject to intentional or unintentional distortion factors, the will of individuals to be truthful in their responses, or the fact that the participants want to be seen as having good and healthy eating habits. However, as far as we know, to date, there are no alternative standardized methods to avoid those issues in nutrition epidemiological studies. Furthermore, as mentioned earlier, we followed the guidance on the EU Menu methodology, as published by the EFSA. This guidance is compulsory for new nutrition epidemiological studies carried out by EU members from 2014 onwards [16]. In addition, we addressed misreporting via a standardized EFSA protocol [12], whereas most epidemiologic studies either ignore misreporting entirely or use inappropriate cut-offs. Therefore, the ANIBES study stands well above many other previous studies on diet quality. 

One main observation in the present study is that older adults had a better diet quality than adults, which was consistently seen when using not only HDI and ANIBES-DQI but also Mediterranean scores. This finding agrees with other reports in Italy [41,42], Greece, and Cyprus [43]. In addition, in other countries like Canada and the USA, using general DQIs, older adults exhibited better diet quality scores than their younger counterparts [8,44]. Thus, North American older adults have higher diet quality scores than adults and youth. However, other factors involved in the socioeconomic status, which may affect the diet quality of the older adults, should be considered. The quality of diet has been related to the socioeconomic status in different populations throughout the world [44,45,46,47], which includes many variables, such as education, income, type of employment, and some characteristics of the particular areas where the populations live. However, there is no unanimity as to how education or the income levels affect diet quality. In the present study, both low education and low income were directly associated with some DQIs, namely the MDS and ANIBES-DQI. Similarly, in a French population, low education was associated with a low-quality diet, characterized by lower intakes of fiber, minerals, and vitamins, although the authors identified very complex interactions among education, income, and occupation (e.g., they described interactions between education and income level) [45]. 

There is a need for more studies to evaluate the relationships between diet quality and socioeconomic status at the domestic level, as diet quality may be influenced by other factors (e.g., the target population, unemployment, the occupation of different members of the family, access to food, urbanization in countries with low or high gross domestic products) [48]. In the ANIBES study, unemployed subjects were included, but we did not gather information about household sizes or single living arrangements. Indeed, we cannot determine the influence of these variables on diet quality. 

The percentages of plausible reporters are essential to determine diet quality for a particular population. In the present study, we found differences between the whole population and the plausible reporters only for the ANIBES-DQI but not for the other DQIs. The influence of misreporting (over- and underreporting) on diet quality has been scarcely considered. Lutomski et al. (2009) observed that under-reporters had a lower diet quality (evaluated by Dietary Approaches to Stop Hypertension (DASH) diet) with a higher nutrient density and a lower percentage of energy derived from fat, whereas over-reporters showed an opposite pattern [48]. The ANIBES study is the only work that has analyzed misreporting in a Spanish population and considered to what extent misreporting could affect diet quality. Thus, it seems that underreporting contributes to increasing the “apparent” percentage of the general population with a low-quality diet. Other factors, including sex, BMI, and education, may also affect the percentages of over and under reporters. However, this difference was not addressed in the present study.

The present study has a number of strengths. Basically, we followed a standardized methodology for the study of nutrient intakes, as well as for the evaluation of misreporting based on EFSA guidelines [9,12,16]. In addition, to study diet quality, we applied selected DQIs that have been validated for global populations, as well as for countries following the Mediterranean pattern [5,21,22,23,24,33]. Moreover, we provided the ANIBES-DQI to evaluate the quality of the diet using cut-off values related to the ARs for the EU. However, this study also has limitations. The first limitation is derived of the use of 24HR, which can contribute to unquantifiable errors, as detailed above, although the use of the CAPI/CATI EFSA methodology would minimize these potential errors [16]. Additional limitations are that there is no gold standard DQI to compare against other indexes. Likewise, it is difficult to compare diet qualities among EU countries from different surveys because of random and systemic errors of diet assessments, due to chronological changes, response rate and linguistic and cultural diversity. Moreover, as previously mentioned, we only stratified DQIs by sex, education, and income, but we did not evaluate other potential factors (e.g., the body mass index of individuals, family size, type of job, unemployment, urbanization, living in rural areas) that could act as effect modifiers.

## 5. Conclusions and Future Research

A high percentage of individuals for the Spanish ANIBES population exhibited elevated intakes of SFA and sugars and did not reach the EU nutrient requirements for some important nutrients like fiber, calcium, zinc, folate, and vitamins A and C. Moreover, low education and low income were associated with low DQIs, and those socioeconomic determinants interacted to produce a lower diet quality. Additionally, the misreported evaluation in the ANIBES population reveals the need to routinely include this analysis in nutrition surveys to give more precise and accurate data related to nutrient intake and diet quality. 

Future research is needed not only in Spain but also in other EU countries to evaluate changes in diet quality in longitudinal, long-term, and well-designed studies with appropriate sizes and regional distribution using the EFSA standardized methodology. In this regard, the use of the ANIBES-DQI may help compare diet quality in EU countries. Furthermore, a deep evaluation of how socioeconomic determinants can affect the diet quality may help in the development of appropriate food and nutrition intervention policies at the regional, country, and global EU levels. 

## Figures and Tables

**Table 1:** Characteristics of the Healthy Diet Indicator (HDI), the Mediterranean-Diet Quality Index (MED-DQI), the Mediterranean Diet Score (MDS), and the Mediterranean Diet Score-modified (MDS-mod).

**HDI (Range 0–9) [21]**			**MED-DQI (Range 0–14) [24]**			
**Components**	**Criteria/Scoring**		**Components**	**Criteria/Scoring**		
	**0**	**1**		**0**	**1**	**2**
Saturated fatty acids (% of total energy)	0–10	>10	Saturated fatty acids (% of total energy)	<10	10–13	>13
Polyunsaturated fatty acids (% of total energy)	3–7	<3 or >7	Cholesterol (mg)	<300	300–400	>400
Protein (% of total energy)	10–15	<10 or >15	Meats (g)	<25	25–125	>125
Complex carbohydrates (% of total energy)	50–70	<50 or >70	Olive oil (ml)	>15	15–5	<5
Dietary fiber (g)	27–40	<27 or >40	Fish (g)	>60	60–30	<30
Fruits and vegetables (g)	>400	<400	Cereals (g)	>300	300–100	<100
Pulses, nuts, seeds (g)	>30	<30	Vegetables and fruits (g)	>700	700–400	<400
Simple Sugars (% of total energy)	0–10	>10				
Cholesterol (mg)	0–300	>300				
**MDS (Range 0–8) [22]**			**MDS-mod (Range 0–9) [23]**			
**Components**	**Criteria/Scoring**		**Components**	**Criteria/Scoring**		
	**0**	**1**		**0**		**1**
Monounsaturated: Saturated fatty acid ratio	<median	>median	Monounsaturated: Saturated fatty acid ratio	<median		≥median
Legumes	<median	>median	Legumes	<median		≥median
Cereals and starchy roots	<median	>median	Cereals	<median		≥median
Fruits and nuts	<median	>median	Fruits and nuts	<median		≥median
Vegetables	<median	>median	Vegetables	<median		≥median
Meat and meat products	>median	<median	Meat	≥median		<median
Milk and dairy products	>median	<median	Fish	<median		≥median
Alcohol	>median	<median	Dairy products	≥median		<median
			Alcohol men	<10–>50		10–50 g/d
			Alcohol women	<5–>25		5–25 g/d

HDI: Huijbregts, 1997 [22]; MDS-DQI: Gerber, 2006 [24]; MDS: Trichopoulou, 1995 [22]; MDS-mod: Trichopolou 2003 [23]; MDS and MDS-mod were adjusted for energy: 2500 kcal for men and 2000 kcal for women.

**Table 2:** ANIBES-DQI.

ANIBES-DQI (Range 0–7)		
Criteria/Scoring		
	0	1
Total Fat	<20% or >35% of the daily energy intake	Between 20% and 35% of the daily energy intake
Saturated fatty acids	>10% of the daily energy intake	≤10% of the daily energy intake
Simple sugars	>10% of the daily energy intake	≤10% of the daily energy intake
Fiber	<AI	≥AI
Vitamin C	<AR	≥AR
Vitamin A	<AR	≥AR
Calcium	<AR	≥AR

ANIBES: Anthropometry, Intake, and Energy Balance Study; AI: Adequate intake; AR: Average requirement.

**Table 3:** Percentage of the ANIBES population not meeting adequate intake recommendations for critical nutrients, stratified by age and sex.

Proteins	Men		Women		Total (%)
Age	AR	%	AR	%	
9–12	1	0.2	1	0.1	0.1
13–17	1	1.6	1	0.6	1.1
18–64	1	11.4	1	4.8	8.1
65–75	1	17.0	1	12.6	14.8
**PUFAs (less than 6% and more than 11% of the total energy reported intake)**					
9–12		24.0		21.8	23.3
13–17		29.9		18.8	25.1
18–64		28.3		21.0	25.4
65–75		27.5		20.1	24.9
**SFAs (more than 10% of the total energy reported intake)**					
9–12		84.1		73.3	80.6
13–17		74.6		76.7	76.5
18–64		63.9		66.4	66.3
65–75		51.0		59.0	56.4
**Simple Sugars (more than 10% of the total energy reported intake)**					
9–12		89.8		94.2	93.2
13–17		85.8		91.1	88.8
18–64		83.4		89.7	86.6
65–75		80.6		93.5	89.8
**Fiber**					
9–12	18	85.1	18	85.7	84.5
13–17	20	93.5	20	98.3	96.0
18–64	25	97.7	25	99.4	98.7
65–75	25	92.1	25	97.5	95.4
**Calcium**					
9–12	820	40.3	820	57.8	46.2
13–17	960	67.6	960	86.6	75.4
18–64	766	62.4	766	69.9	65.2
65–75	750	68.8	750	69.0	67.9
**Iron**					
9–12	8	5.8	8	7.0	6.4
13–17	8	6.0	7	3.9	5.0
18–64	6	0.6	7	2.6	1.6
65–75	6	0.8	7	2.7	1.8
**Zinc**					
9–12	8	26.6	8	41.0	31.3
13–14	11	71.3	10	78.6	76.9
**Folate**					
9–12	185	55.8	185	62.4	61.8
13–17	234	80.0	234	92.6	84.9
18–64	250	85.5	250	91.1	88.3
65–75	250	84.8	250	83.4	81.8
**Vitamin C**					
9–12	50	37.4	50	34.0	35.7
13–17	75	60.0	69	65.3	62.6
18–64	90	56.3	80	49.4	52.9
65–75	90	37.1	80	23.1	30.1
**Vitamin A**					
9–12	400	18.6	400	27.6	23.1
13–17	540	38.6	486	52.2	45.4
18–64	570	41.9	490	38.7	40.3
65–75	570	40.4	490	28.1	34.3

ANIBES: Anthropometry, Intake and Energy Balance Study; PUFAs: Polyunsaturated fatty acids; SFAs: Saturated fatty acids; AR: Average requirement.

**Table 4:** Percentage of the ANIBES population according to diet quality indexes (DQIs), classified by cut-off values in adults and older adults and stratified by sex.

					Adults 18–64 Years			Older Adults 65–75 Years		
		Total	Men	Women	Total	Men	Women	Total	Men	Women
**HDI**	Low	59.0	61.2	57.0	60.2	63.0	57.6	49.0	46.5	51.4
	Medium	35.6	33.6	37.4	34.9	32.2	37.5	40.8	44.4	37.4
	High	5.4	5.2	5.6	4.8	4.8	4.9	10.2	9.1	11.2
**MDS**	Low	44.8	37.9	51.1	47.4	40.5	53.8	23.8	17.2	29.9
	High	55.2	62.1	48.9	52.6	59.5	46.2	76.2	82.8	70.1
**MDS-mod**	Low	33.2	32.1	34.2	35.5	34.5	36.5	14.6	13.1	15.9
	Medium	42.3	42.3	42.4	41.6	41.6	41.7	48.1	47.5	48.6
	High	24.4	25.6	23.3	22.8	23.9	21.8	37.4	39.4	35.5
**MED-DQI**	Poor	9.3	10.4	8.4	10.0	11.4	8.8	3.9	2.0	5.6
	Medium Poor	39.9	41.7	38.3	41.8	44.0	39.7	25.2	23.2	27.1
	Medium Good	39.6	39.2	39.9	38.5	37.2	39.7	48.5	55.6	42.1
	Good	11.1	8.7	13.4	9.7	7.4	11.9	22.3	19.2	25.2
**ANIBES**	Low	69.5	68.5	70.5	70.7	69.0	72.2	60.2	63.6	57.0
	Medium	26.2	26.4	25.9	25.4	26.3	24.6	32.0	27.3	36.4
	High	4.3	5.1	3.5	3.9	4.6	3.2	7.8	9.1	6.5

ANIBES: Anthropometry, Intake and Energy Balance Study; HDI: Healthy Diet Indicator; MDS: Mediterranean Diet Score; MDS-mod: Modified Mediterranean Diet Score; MED-DQI: Mediterranean Diet Score.

**Table 5:** Percentages of the ANIBES population and associations of education and income levels and their interactions with the degree of diet quality for each diet quality score.

		Education			Income (Euros/Month)			*p* Value (Kendal’s Tau-c Coefficient)		
		Primary	Secondary	Tertiary	<1000	1000–2000	>2000	Education	Income	Interaction
**HDI**	Low	18.7	27.1	12.9	15.2	30.4	13.1	0.503 (−0.670)	0.387 (−0.887)	0.380 (−0.874)
	Medium	12.3	14.8	8.7	10.8	17.2	7.8			
	High	2.1	2.2	1.3	1.4	3.0	1.2			
**MDS**	Low	12.7	21.9	10.4	40.4	10.9	18.7	**0.011** (−2.548)	**0.015** (−2.443)	**0.004** (−2.864)
	High	20.3	22.2	12.5	52.3	23.4	10.7			
**MDS-mod**	Low	10.1	16.3	6.5	30.8	27.1	11.4	0.826 (−0.220)	0.440 (−0.772)	0.599 (−0.526)
	Medium	14.9	17.6	10.1	39.7	17.2	7.4			
	High	8.1	10.1	6.2	22.2	21.8	9.0			
**MED-DQI**	Poor	2.6	5.3	1.5	8.9	11.4	5.7	0.143 (−1.464)	0.397 (−0.847)	0.325 (−0.526)
	Medium Poor	8.7	17.9	9.5	32.8	5.1	2.0			
	Medium Good	14.1	17.0	9.1	37.2	16.2	9.2			
	Good	4.0	30.4	3.0	10.1	20.6	8.4			
**ANIBES**	Low	21.1	30.0	13.8	62.1	4.9	2.6	0.270 (−1.103)	**0.010** (−2.566)	**0.028** (−2.197)
	Medium	10.6	11.3	7.7	26.4	32.8	13.4			
	High	1.3	2.4	1.3	4.2	14.9	7.5			

ANIBES: Anthropometry, Intake and Energy Balance Study; HDI: Healthy Diet Indicator; MDS: Mediterranean Diet Score; MDS-mod: Modified Mediterranean Diet Score; MED-DQI: Mediterranean Diet Score. *p* values correspond to the significance of the associations of education and income levels and their interactions with the degree of diet quality for each DQI, estimated by Kendal’s Tau-c rank correlation coefficients, in parentheses. Figures in bold are: statistically significant.

## Supplementary Materials

The following are available online at https://www.mdpi.com/2072-6643/11/10/2328/s1, Table S1: Percentage of the ANIBES population for each diet quality index (DQI) stratified by education and income levels, Table S2: Major estimated effects of education and income on the degree of diet quality for each DQI based on ordinal regression general linear models, Table S3: Percentage of the ANIBES population (AP) and the plausible energy reporters (PER) for each diet quality index (DQI).
