# Supplementary material for: Adequacy of Critical Nutrients Affecting the Quality of the Spanish Diet in the ANIBES Study
Source: Nutrients. 2019 Oct 1;11(10):2328. doi: 10.3390/nu11102328 (PMC6835880; doi:10.3390/nu11102328)
Supplement: Supplementary file 1 [file nutrients-11-02328-s001.pdf]

Supplementary Materials: Adequacy of Critical Nutrients Affecting the Quality of the Diet in the ANIBES Study

Josune Olza[1,2,3], Emilio Martínez de Victoria[4], Javier Aranceta-Bartrina[3,5], Marcela González-Gross[3,6], Rosa M. Ortega[7], Lluis Serra-Majem[3,8], Gregorio Varela-Moreiras[9,10] and Ángel Gil[1,2,3,]**

Supplementary Table 1. Percentage of the ANIBES population for each diet quality index (DQI) stratified by education and income levels.

DQIs		**Education**									
		Primary			*Secondary*			*Tertiary*			
	**Income (euros/month)**	**<1000**	**1000–2000**	**>2000**	**<1000**	**1000–2000**	**>2000**	**<1000**	**1000–2000**	**>2000**	**Total**
HDI	Low	8.4	8.6	1.7	5.3	15.1	6.7	1.5	6.7	4.7	58.7
	Medium	6.0	5.3	1.0	3.4	8.0	3.4	1.4	3.9	3.4	35.8
	High	1.2	0.8	0.1	0.1	1.5	0.6	0.1	0.7	0.5	5.6
	Total	15.6	14.7	2.8	8.8	24.6	10.7	3.0	11.3	8.6	
MDS	Low	5.8	5.8	1.1	3.9	12.6	5.4	1.2	5.0	4.2	45
	High	9.8	8.8	1.7	4.9	12.0	5.3	1.8	6.3	4.4	55
	Total	15.6	14.6	2.8	8.8	24.6	10.7	3.0	11.3	8.6	
MDS-MOD	Low	4.4	4.6	1.1	3.2	8.9	4.2	0.7	3.7	2.1	32.9
	Medium	7.2	6.7	1.0	3.2	10.7	3.7	1.4	4.4	4.3	42.6
	High	4.0	3.3	0.8	2.4	5.0	2.7	0.9	3.1	2.2	24.4
	Total	15.6	14.6	2.9	8.8	24.6	10.6	3.0	11.2	8.6	
MED-DQI	Poor	1.0	1.3	0.3	1.0	3.1	1.2	0.3	0.7	0.5	9.4
	Medium poor	6.2	5.1	1.1	3.4	10.1	4.4	1.1	4.7	3.7	39.8
	Medium good	6.1	6.8	1.2	3.7	9.2	4.1	1.4	4.6	3.1	40.2
	Good	2.3	1.4	0.3	0.7	2.2	0.9	0.3	1.3	1.4	10.8
	Total	15.6	14.6	2.9	8.8	24.6	10.6	3.1	11.3	8.7	
ANIBES-DQI	Low	10.6	8.8	1.7	6.5	16.8	7.1	2.0	7.2	4.6	65.3
	Medium	4.5	5.1	1.0	1.8	6.4	3.1	0.9	3.4	3.4	29.6
	High	0.5	0.7	0.1	0.4	1.5	0.5	0.1	0.6	0.6	5.0
	Total	15.6	14.6	2.8	8.7	24.7	10.7	3.0	11.2	8.6	

ANIBES: Anthropometry. Intake and Energy Balance Study; HDI: Healthy Diet Indicator; MDS: Mediterranean Diet Score; MDS-MOD: Mediterranean Diet Score-modified; MED-DQI: Mediterranean-Diet Quality Index; ANIBES-DQI: ANIBES-Diet Quality Index .

Supplementary Materials: Adequacy of Critical Nutrients Affecting the Quality of the Diet in the ANIBES Study

Josune Olza[1,2,3], Emilio Martínez de Victoria[4], Javier Aranceta-Bartrina[3,5], Marcela González-Gross[3,6], Rosa M. Ortega[7], Lluis Serra-Majem[3,8], Gregorio Varela-Moreiras[9,10] and Ángel Gil[1,2,3, **]

Supplementary Table 2.Major estimated effects of education and income on the degree of diet quality for each DQI based on ordinal regression general linear models.

DQI	Category	Covariates	Effect (x)	e^{-x}	p value
**HDI[a]**	Low	**Education**			
		Primary	-0.196	1.217	0.554
		Secondary	0.194	0.823	0.534
		Tertiary	0[b]		
		**Income**			
		<1000	0.166	0.847	0.656
		1000–2000	-0.056	1.057	0.855
		>2000	0[b]		
	Medium	**Education**			
		Primary	-0.288	1.333	0.398
		Secondary	-0.033	1.034	0.919
		Tertiary	0[b]		
		**Income**			
		<1000	0.338	0.713	0.375
		1000–2000	-0.089	1.093	0.778
		>2000	0[b]		
**MDS[a]**	Low	**Education**			
		Primary	**0.455**	**0.634**	**0.000**
		Secondary	**0.369**	**1.446**	**0.020**
		Tertiary	0[b]		
		**Income**			
		<1000	**0.359[1]**<br>**0.268[2]**	**0.698**<br>**0.765**	**0.019**<br>**0.035**
		1000–2000	0.091	0.913	0.503
		>2000	0[b]		
	Medium	**Education**			
		Primary	0.042	1.043	0.789
		Secondary	0.019	1.020	0.897
		Tertiary	0[b]		
		**Income**			
		<1000	0.028	1.029	0.881
		1000–2000	0.207	1.230	0.2287
		>2000	0[b]		
**MDS-mod[a]**	Low	**Education**			
		Primary	0.237	0.789	0.249
		Secondary	**0.439**	**0.645**	**0.017**
		Tertiary	0[b]		
		**Income**			
		<1000	-0.145	1.156	0.496
		1000–2000	0.130	0.878	0.474
		>2000	0[b]		
	Medium	**Education**			
		Primary	0.146	0.864	0.443
		Secondary	0.065	0.937	0.707
		Tertiary	0[b]		
		**Income**			
		<1000	-0.028	1.028	0.892
		1000–2000	0.182	0.834	0.297

		>2000	0[b]		
**MED-DQI[c]**	Poor	**Education**			
		Primary	0.104[1]	0.901	0.379
			**0.376[2]**	**0.687**	**0.000**
		Secondary	**-0.275**	**1.317**	**0.013**
		Tertiary	0[b]		
		**Income**			
		<1000	-0.106	0.900	0.756
		1000–2000	0.321	1.378	0.287
		>2000	0[b]		
	Medium Poor	**Education**			
		Primary	**0.376**	**0.687**	**0.000**
		Secondary	0.275	1.317	0.171
		Tertiary	0[b]		
		**Income**			
		<1000	-0.089	0.914	.721
		1000–2000	0.140	1.150	0.540
		>2000	0[b]		
	Medium Good	**Education**			
		Primary	**0.376**	**0.687**	**0.000**
		Secondary	0.114	1.120	0.574
		Tertiary	0[b]		
		**Income**			
		<1000	0.048	1.049	0.847
		1000–2000	0.255	1.290	0.268
		>2000	0[b]		
**ANIBES[a]**	Low	**Education**			
		Primary	-0.168	1.183	0.252
		Secondary	**-0.353**	**1.423**	**0.012**
		Tertiary	0[b]		
		**Income**			
		<1000	**-0.396**	**1.486**	**0.012**
		1000–2000	-0.173	1.189	0.209
		>2000	0[b]		

ANIBES: Anthropometry, Intake and Energy Balance Study; HDI: Healthy Diet Indicator; MDS: Mediterranean Diet Score; MDS-mod: Modified Mediterranean Diet Score; MED: Mediterranean x x: Diet Score. ordinal regression coefficient; e^-x: probability of significant effects of the covariates (income and education) on different DQI categories. Significant values are highlighted in bold

[a]. The reference category is: High

[b]. This parameter is set to zero because it is redundant.

[c]. The reference category is: Good.

[1] Primary vs. tertiary

[2] Primary vs. secondary

Supplementary Materials: Adequacy of Critical Nutrients Affecting the Quality of the Diet in the ANIBES Study

Josune Olza[1,2,3], Emilio Martínez de Victoria[4], Javier Aranceta-Bartrina[3,5], Marcela González-Gross[3,6], Rosa M. Ortega[7], Lluis Serra-Majem[3,8], Gregorio Varela-Moreiras[9,10] and Ángel Gil[1,2,3,]**

Supplementary Table 3. Percentage of the ANIBES population (AP) and the plausible energy reporters (PER) for each diet quality index (DQI)

			Total			Adults (18–64 years)			Older adults (65–75 years)		
			Total	Men	Women	Total	Men	Women	Total	Men	Women
HDI	AP	Low	59.0	61.2	57.0	60.2	63.0	57.6	49.0	46.5	51.4
		Medium	35.6	33.6	37.4	34.9	32.2	37.5	40.8	44.4	37.4
		High	5.4	5.2	5.6	4.8	4.8	4.9	10.2	9.1	11.2
	PER	Low	58.6	61.4	55.5	59.8	63.1	56.2	49.1	46.7	51.2
		Medium	36.7	34.3	39.4	36.1	33.0	39.5	41.6	45.3	38.4
		High	4.7	4.3	5.1	4.1	3.9	4.3	9.3	8.0	10.5
MDS	AP	Low	44.8	37.9	51.1	47.4	40.5	53.8	23.8	17.2	29.9
		High	55.2	62.1	48.9	52.6	59.5	46.2	76.2	82.8	70.1
	PER	Low	40.7	35.2	46.6	43.1	37.7	49.1	22.4	14.7	29.1
		High	59.3	64.8	53.4	56.9	62.3	50.9	77.6	85.3	70.9
MDS-MOD	AP	Low	33.2	32.1	34.2	35.5	34.5	36.5	14.6	13.1	15.9
		Medium	42.3	42.3	42.4	41.6	41.6	41.7	48.1	47.5	48.6
		High	24.4	25.6	23.3	22.8	23.9	21.8	37.4	39.4	35.5
	PER	Low	36.5	35.0	38.2	38.5	36.9	40.4	21.1	18.7	23.3
		Medium	41.7	42.5	40.9	41.5	42.3	40.5	43.5	44.0	43.0
		High	21.8	22.5	21.0	20.0	20.8	19.1	35.4	37.3	33.7
MED-DQI	AP	Poor	9.3	10.4	8.4	10.0	11.4	8.8	3.9	2.0	5.6
		Medium Poor	39.9	41.7	38.3	41.8	44.0	39.7	25.2	23.2	27.1
		Medium Good	39.6	39.2	39.9	38.5	37.2	39.7	48.5	55.6	42.1
		Good	11.1	8.7	13.4	9.7	7.4	11.9	22.3	19.2	25.2
	PER	Poor	8.3	10.5	6.0	8.9	11.6	6.0	3.7	1.3	5.8
		Medium Poor	40.6	41.7	39.4	42.4	43.4	41.2	26.7	26.7	26.7
		Medium Good	40.6	39.4	41.9	39.7	37.5	42.1	47.8	56.0	40.7
		Good	10.5	8.4	12.7	9.0	7.5	10.7	21.7	16.0	26.7
		Best	0.0	0.0	0.0	0.0	0.0	0.0	0.0	0.0	0.0
ANIBES-DQI	AP	Low	69.5	68.5	70.5	70.7	69.0	72.2	60.2	63.6	57.0
		Medium	26.2	26.4	25.9	25.4	26.3	24.6	32.0	27.3	36.4
		High	4.3	5.1	3.5	3.9	4.6	3.2	7.8	9.1	6.5
	PER	Low	49.0	43.4	52.4	50.8	45.6	53.8	31.1	29.2	33.3
		Medium	41.2	40.1	41.9	40.2	39.2	40.7	51.1	45.8	57.1

High	9.8	15.5	5.7	9.0	15.2	5.5	17.8	25.0	9.5

ANIBES: Anthropometry, Intake and Energy Balance Study; HDI: Healthy Diet Indicator; MDS: Mediterranean Diet Score; MDS-mod: Modified Mediterranean Diet Score; MED-DQI: Mediterranean Diet Score; ANIBES-DQI: ANIBES-Diet Quality Index
